# Classifying Patient Complaints Using Artificial Intelligence–Powered Large Language Models: Cross-Sectional Study

**DOI:** 10.2196/74231

**Published:** 2025-08-06

**Authors:** Sky Wei Chee Koh, Eunice Rui Ning Wong, John Chong Min Tan, Stephanie C C van der Lubbe, Jun Cong Goh, Ethan Sheng Yong Ching, Ian Wen Yih Chia, Si Hui Low, Ping Young Ang, Queenie Quek, Mehul Motani, Jose M Valderas

**Affiliations:** 1 Division of Family Medicine, Department of Medicine, Yong Loo Lin School of Medicine, National University of Singapore, NUHS Tower Block Level 9, 1E Kent Ridge RoadSingapore, 119228, Singapore, 65 67163185; 2 National University Polyclinics, National University Health System, Singapore, Singapore; 3 Department of Family Medicine, National University Health System, Singapore, Singapore; 4 Department of Electrical and Computer Engineering, College of Design and Engineering, National University of Singapore, Singapore, Singapore; 5 Institute of Data Science, N.1 Institute for Health, Institute for Digital Medicine, National University of Singapore, Singapore, Singapore; 6 Centre for Research in Health Systems Performance and Department of Medicine, Yong Loo Lin School of Medicine, National University of Singapore, Singapore, Singapore

**Keywords:** artificial intelligence, patient complaints, primary care, large language models, health services, family medicine

## Abstract

**Background:**

Patient complaints provide valuable insights into the performance of health care systems, highlighting potential risks not apparent to staff. Patient complaints can drive systemic changes that enhance patient safety. However, manual categorization and analysis pose a huge logistical challenge, hindering the ability to harness the potential of these data.

**Objective:**

This study aims to evaluate the accuracy of artificial intelligence (AI)–powered categorization of patient complaints in primary care based on the Healthcare Complaint Analysis Tool (HCAT) General Practice (GP) taxonomy and assess the importance of advanced large language models (LLMs) in complaint categorization.

**Methods:**

This cross-sectional study analyzed 1816 anonymous patient complaints from 7 public primary care clinics in Singapore. Complaints were first coded by trained human coders using the HCAT (GP) taxonomy through a rigorous process involving independent assessment and consensus discussions. LLMs (GPT-3.5 turbo, GPT-4o mini, and Claude 3.5 Sonnet) were used to validate manual classification. Claude 3.5 Sonnet was further used to identify complaint themes. LLM classifications were assessed for accuracy and consistency with human coding using accuracy and *F*_1_-score. Cohen κ and McNemar test evaluated AI-human agreement and compared AI models’ concordance, respectively.

**Results:**

The majority of complaints fell under the HCAT (GP) domain of management (1079/1816, 59.4%), specifically relating to institutional processes (830/1816, 45.7%). Most complaints were of medium severity (994/1816, 54.7%), occurred within the practice (627/1816, 34.5%), and resulted in minimal harm (75.4%). LLMs achieved moderate to good accuracy (58.4%‐95.5%) in HCAT (GP) field classifications, with GPT-4o mini generally outperforming GPT-3.5 turbo, except in severity classification. All 3 LLMs demonstrated moderate concordance rates (average 61.9%‐68.8%) in complaints classification with varying levels of agreement (κ=0.114‐0.623). GPT-4o mini and Claude 3.5 significantly outperformed GPT-3.5 turbo in several fields (*P*<.05), such as domain and stage of care classification. Thematic analysis using Claude 3.5 identified long wait times (393/1816, 21.6%), staff attitudes (287/1816, 15.8%), and appointment booking issues (191/1816, 10.5%) as the top concerns, which accounted for nearly half of all complaints.

**Conclusions:**

Our study highlighted the potential of LLMs in classifying patient complaints in primary care using HCAT (GP) taxonomy. While GPT-4o and Claude 3.5 demonstrated promising results, further fine-tuning and model training are required to improve accuracy. Integrating AI into complaint analysis can facilitate proactive identification of systemic issues, ultimately enhancing quality improvement and patient safety. By leveraging LLMs, health care organizations can prioritize complaints and escalate high-risk issues more effectively. Theoretically, this could lead to improved patient care and experience; further research is needed to confirm this potential benefit.

## Introduction

Patients are a valuable source of information regarding the current state of the health care system [1], uniquely positioned to identify potential risks not apparent to staff. Their complaints contribute meaningfully to quality improvement and safety, driving systemic changes that enhance patient care, as evidenced by initiatives such as patient feedback questionnaires in the United Kingdom [2] and patient harm measurement in Canadian hospitals [3].

In recent years, the Healthcare Complaint Analysis Tool (HCAT) has facilitated the examination of complaints in health care, providing valuable insights into complaint types, severity, and patient harm across various stages of care [4,5]. By identifying “hot spots” (areas prone to harm or near misses) and “blind spots” (areas overlooked by staff), organizations can take targeted actions to improve care [6]. Although the HCAT has demonstrated validity, reliability, and adaptability across diverse health care systems, languages, and contexts—including primary care settings (HCAT General Practice [GP]) [7-9]—the sheer volume of complaints, combined with the complexity of manual categorization and analysis, poses a significant obstacle. This bottleneck hinders the ability to harness the full potential of this valuable data. As a result, addressing complaints in health care often remains a reactive process, focusing on resolving individual issues rather than identifying and addressing systemic challenges [10,11].

Recent advancements in artificial intelligence (AI), specifically AI-powered natural language processing (NLP) through large language models (LLM), offer a promising solution to efficiently and accurately classify complaints, automating labor-intensive processes and freeing up resources for strategic quality improvement initiatives [12-14]. While some applications of LLM to text data appear indeed promising, the performance of existing approaches in the health care domain has been relatively lower, achieving around 80% accuracy [15]. Various studies have used different methods to improve accuracy, such as sentiment analysis [16] and NLP algorithms [17,18], highlighting the need for further refinement and domain-specific adaptation to accurately capture the nuances of health care–related complaints. The advent of advanced LLM and ongoing refinements to existing architectures have also sparked interest in exploring the feasibility of leveraging AI for day-to-day complaints analysis without further model training. Harnessing the potential of LLMs, such as OpenAI’s GPT and Anthropic’s Claude, the complaint analysis process can be automated, ultimately enhancing the efficiency and effectiveness of quality improvement initiatives in primary care.

This study aims to evaluate the accuracy of LLM in categorization of patient complaints in primary care according to the HCAT (GP) taxonomy. We also aim to assess whether the newer LLM technologies have improved accuracy in categorization.

## Methods

### Study Design

We conducted a cross-sectional data extraction of patient feedback complaints in public primary care providers in Singapore from the unified feedback management system for 7 public primary care clinics (January 01, 2021, to December 31, 2021). Patient complaints refer to the patients’ feedback expressing their dissatisfaction or concern about the care quality, service, or experience during their care journey. The patient feedback was collected from various channels, including electronic mail, website, phone, physical feedback forms, and relevant authorities. The feedback management system was managed by Service Quality staff, who labeled and categorized feedback daily. For non-English complaints or verbatim accounts collected, these were translated into English using the Microsoft Translator tool integrated into Microsoft Outlook on Windows. The Service Quality staff used predefined categories to tag feedback as either compliments, complaints, or issues unrelated to primary health care. We first included identified complaints into the analysis. We then manually reviewed feedback initially tagged as compliments or unrelated issues to identify any hidden complaints. Eligible feedback, including instances where feedback contained both compliments and complaints, was subsequently included in the analysis.

### Measurements

The HCAT taxonomy was developed through a rigorous process, which included literature review, validation, and reliability testing [5], resulting in a theoretically informed and reliable tool for coding health care complaints [19,20]. The HCAT (GP) adaptation was then developed and demonstrated good validity for coding patient complaints in GP settings [7]. We used the HCAT (GP) taxonomy (Multimedia Appendix 1) adapted from the study by O’Dowd et al [7] to code complaint domain, categories, severity, stage of care, and level of harm. For the purposes of this study, we aligned our definitions and terminology with those established by the HCAT (GP) framework. Four trained coders (2 physicians, SWCK and ERNW, and 2 medical students, ESYC and IWHC), familiar with the HCAT handbook and online training, conducted manual categorization [21]. To ensure inter-rater reliability, coders reviewed a sample of 30 complaints together to establish consistency in coding. Each coder then independently assessed preassigned batches of complaints, with each complaint reviewed by 2 coders. In cases of disagreement, a third coder was involved to categorize the complaint, and any remaining discrepancies were resolved through consensus discussions.

### Data Cleaning and Analysis

Data cleaning and analysis were performed using Rstudio (R version 4.2.0; Posit, PBC), IBM SPSS Statistics (version 29.0; IBM Corp), and Microsoft Excel 2010. Descriptive statistics, prevalence, and characteristics of complaints were analyzed. We calculated the incidence of complaints as the number of complaints per 100,000 attendances per year, to allow for comparison with other complaints studies.

### LLM Configuration and Prompt Design for Complaint Classification

We then evaluated the accuracy of 2 LLMs, GPT and Claude, in patient complaints classification, benchmarking their performance against our manual HCAT (GP) classification. For the purposes of this study, we used GPT-4o mini and Claude 3.5 Sonnet and compared GPT-4o mini with its predecessor, GPT-3.5 turbo. All 1816 patient complaints were included for LLM classification.

After de-identification of patient complaints, we used the StrictJSON library [22] to interface with OpenAI’s GPT-3.5 turbo and GPT-4o mini, as well as Claude 3.5 Sonnet via API using Python. Given the historical token length limitations of LLMs (eg, GPT-3.5’s 8000 input token limit) and the need for the system to handle arbitrarily long complaints, we found that the majority (97.4%) of our complaints were 3000 characters or less. For complaints exceeding 3000 characters, we truncated them to the first 3000 characters. The models were used in their off-the-shelf configurations without any fine-tuning or modifications to their architectures or pipelines. Prompts were done singly for each complaint. We provided the LLM with these complaints and the original HCAT (GP) description as context, asking it to output the closest classification for each complaint in a multinomial classification framework, where the primary issue was identified as the most viable classification. To accurately classify severity, which is context-specific and varies in description and level across categories, we conditioned our approach on the category using ground truth information and supplemented this with HCAT (GP) severity keywords to enhance classification results, thereby leveraging category-specific knowledge to inform severity assessments.

The prompts were designed to fit the specific HCAT fields by incorporating the category descriptions and keywords. For each field, we provided a clear definition and inputted the variables with word-for-word descriptions in accordance with HCAT (GP) (Textbox 1).

Textbox 1. **Patient complaint classification framework using large language models.**

- For the “Domain” field, we defined it to the LLM as: “Domain refers to the problem domain of which the complaint is referring to.”
- We then inputted the variables within “Domain” with the following description: “Clinical Problems: Issues relating to quality and safety of clinical and nursing care provided by healthcare staff. Keywords: ‘not provided,’ ‘was not done,’ ‘did not follow guidelines,’ ‘poor standards,’ ‘should have,’ ‘not completed,’ ‘unacceptable quality,’ ‘not successful,’ ‘incorrect,’ ‘medication error,’ ‘did not notice,’ ‘mistake,’ ‘failed to act,’ ‘wrong,’ ‘poor coordination,’ ‘unaware,’ ‘missed the signs,’ ‘diagnosis.’”
- The above was then populated in all the other fields below:

- “You are a classifier to sort patient complaints from a hospital visit into categories based on {category_name}.
- Patient Complaint: {text}
- Description of category: {zero_shot_description}
- Category Letter - Description: {category_description}”
- Where {category_name}, {text}, {zero_shot_description}, and {category_description} contained the list of available categories, the de-identified complaint, the .. We then ask the LLM to output in the following JSON (JavaScript Object Notation) format: {“Summary of Patient Complaint in one sentence”: “Summary,” “Classification”: “Category Letter”}

We used a chain-of-thought methodology, where the LLM first summarizes the patient complaint to facilitate better processing, and then uses this summary to generate the classification. We used a bare-bones approach using in-context LLM prompting, using off-the-shelf models without architecture or pipeline modifications. Our classification models were not fine-tuned on ground truth data and instead relied on the models’ pretrained knowledge and the provided context. The prompts used for the LLMs and full codes are provided in Multimedia Appendix 1.

### Evaluation Metrics for LLM Performance in Complaints Classification

To assess the effectiveness of LLM, we used a set of evaluation metrics to provide an understanding of the LLMs’ performance, using our human-determined classification as the benchmark. These measures were used in a previous study to evaluate LLM classification of patient complaints [17].

Accuracy measures the overall correctness of LLM classifications: Accuracy = (True Positives+True Negatives) / (True Positives+False Positives+True Negatives+False Negatives).

*F*_1_-score provides a balanced measure of the LLMs’ accuracy, considering both precision and recall: *F*_1_-score=2×(Precision×Recall) / (Precision+Recall).

We excluded complaints categorized as major or catastrophic harm due to insufficient sample size, ensuring more reliable calculations of the above metrics.

We then conducted an inter-rater agreement analysis to evaluate the consistency between the LLMs’ predictions and our manual HCAT (GP) classification. Specifically, we calculated (1) percentage agreement: the proportion of instances where the LLMs’ predictions matched the manual classification, providing an overall measure of concordance, which is the degree of similarity between 2 classification sets; and (2) Cohen Kappa (κ): a specific measure that evaluates the accuracy (degree of correctness) and reliability (consistency of classification) of the LLMs’ predictions, which accounts for chance agreements.

Finally, we conducted a thematic classification of patient complaints using Claude 3.5 Sonnet into distinct topics. We instructed the LLM to generate a taxonomy comprising 15 nonredundant categories for complaint classification. The selection of 15 categories was informed by our internal feedback management system, which uses a similar categorization framework consisting of 15 distinct categories (eg, waiting time and communication). This approach enabled us to also investigate the congruence between the AI-generated categories and our existing internal framework.

### Ethical Considerations

The research was conducted in accordance with the Declaration of Helsinki national and institutional standards and approved by the National Healthcare Group Domain-Specific Review Board on September 22, 2022 (2022/00333). A waiver of consent was granted for this study as it was deemed to be of minimal risk to the participants involved. Data were approved for use by the relevant institutional approving bodies, extracted and analyzed as per institutional guidelines and policies.

## Results

### Overview

A total of 1816 complaints were made, out of 1,680,828 primary care physician attendances in 2021, yielding a complaint rate of 108 per 100,000 attendances per year. In total, 67.8% (1232/1816) of these complaints were submitted electronically (email or website), with the majority (1308/1816, 72.0%) made by patients themselves and mostly related to operations (1029/1816, 56.7%; Table 1).

**Table 1.** Description of complaint characteristics and HCAT (GP)^a^ manual classification.

Complaint characteristics	Values, n (%)
Complaint origin	
Patient	1308 (72.0)
Family member	462 (25.4)
Others	46 (2.5)
Complaints distribution by departments	
Operations	1029 (56.7)
Medical	249 (13.7)
Headquarters^b^	197 (10.8)
Nursing	125 (6.9)
Diagnostics	77 (4.2)
Dental	76 (4.2)
Pharmacy	50 (2.8)
Allied health^c^	13 (0.7)
Complaints as manually classified using HCAT (GP)	1816 (100)
Clinical	318 (17.5)
Quality (clinical standards of staff and behavior)	205 (11.3)
Safety (errors, incidents, and staff competencies)	113 (6.2)
Management	1079 (59.4)
Environment (facilities, services, equipment, staffing levels)	249 (13.7)
Institutional processes (bureaucracy, wait time, accessing care)	830 (45.7)
Relationship	419 (23.1)
Listening (disregarded or unacknowledged patient information)	61 (3.4)
Communication (absent or incorrect communication from staff)	98 (5.4)
Respect and patient rights (disrespect or violations by staff)	260 (14.3)
Severity of complaints	
Low	444 (24.4)
Medium	994 (54.7)
High	378 (20.8)
Stage of care	
Accessing care	465 (25.6)
While in the practice	627 (34.5)
During the consultation	383 (21.1)
Referral or follow-up	201 (11.1)
Unspecified or Other	140 (7.7)
Patient harm	
No harm	105 (5.8)
Minimal harm	1369 (75.4)
Minor harm	257 (14.2)
Moderate harm	84 (4.6)
Major harm	1 (0.1)
Catastrophic harm	0 (0)

a HCAT (GP): Healthcare Complaint Analysis Tool (General Practice).

b Headquarter departments consist of clinical informatics, clinical services, communications, contact center, finance, human resources, and operations support.

c Allied health professionals consist of dieticians, medical social workers, physiotherapists, podiatrists, and psychologists.

In terms of the HCAT (GP) domain, most of the complaints (1079/1816, 59.4%) were management related, followed by relationship (419/1816, 23.1%) then clinical (318/1816, 17.5%). Within complaints that were management-related, a sizeable proportion was attributed to institutional processes (830/1816, 45.7%) (Table 1). In relation to complaint severity classified according to HCAT (GP), most complaints were categorized as medium severity (994/1816, 54.7%). High numbers of complaints occurred while in the practice (627/1816, 34.5%) and while accessing care (465/1816, 25.6%). Overall, 75.4% (2369/1816) of complaints resulted in minimal harm, while only one complaint resulted in major harm (Table 1).

### Evaluating LLM Performance in Complaints Classification

The performance of LLMs in classifying complaints according to HCAT (GP) fields is presented in Table 2. Notably, all LLMs achieved good accuracy in domain classification (84.5%-88.3%), with particularly strong *F*_1_-scores in the management domain (0.87-0.88). In addition, the models demonstrated fair to excellent accuracy in classifying complaints by stage of care (74.9%-94.1%). In comparison, GPT-4o mini generally outperformed its predecessor, GPT-3.5 turbo, in most HCAT (GP) fields, including domain, stage of care, and patient harm. However, GPT-3.5 turbo surprisingly had higher *F*_1_-scores in classifying complaints severity compared to GPT-4o mini.

**Table 2.** Comparative analysis of large language models in complaints classification: accuracy and *F*_1_-score.

HCAT (GP)^a^ fields	Accuracy			*F*_1_-score		
	GPT3.5	GPT4o	Sonnet	GPT3.5	GPT4o	Sonnet
Domain						
Clinical	86.8	86.8	88.3^b^	0.58	0.58^b^	0.53
Management	84.9	85.0^b^	84.5	0.87	0.88	0.88^b^
Relationship	85.0	86.8	88.2^b^	0.70	0.71	0.73^b^
Severity						
Low	58.4	69.8^b^	60.4	0.46	0.39	0.48^b^
Medium	61.7	51.7	63.4^b^	0.59	0.54	0.61^b^
High	77.6	65.7	78.9^b^	0.25	0.24	0.29^b^
Stage of care						
Accessing care	81.3	85.5	86.5^b^	0.66	0.71	0.71^b^
While in the practice	74.9	77.3	79.0^b^	0.64	0.73	0.75^b^
During the consultation	85.7	89.5	90.7^b^	0.69	0.69	0.75^b^
Referral or follow-up	87.3^b^	86.0	86.6	0.24	0.31	0.40^b^
Unspecified or other	92.2	94.1^b^	93.5	0.32	0.53^b^	0.31
Patient harm						
No harm	73.0	92.6^b^	91.9	0.16	0.15	0.30^b^
Minimal harm	61.1	76.7^b^	76.6	0.73	0.86^b^	0.86^b^
Minor harm	85.6	85.4	86.1^b^	0.29^b^	0.08	0.13
Moderate harm	95.5^b^	94.1	95.5^b^	0.28	0.31^b^	0.15

a HCAT (GP): Healthcare Complaint Analysis Tool (General Practice).

b These values showcase the highest values within the row.

### Concordance and Agreement in Complaints Classification

All 3 LLMs demonstrated overall moderate concordance (61.9%‐68.8%) in classifying HCAT (GP) fields (Table 3). They achieved high concordance in classifying patient complaints according to their HCAT (GP) domains, achieving 78.4% for GPT-3.5 turbo, 79.4% for GPT-4o mini, and 80.5% for Claude 3.5 Sonnet, respectively, with substantial agreement (GPT-3.5 turbo: κ=0.612, GPT-4o mini: κ=0.623, Claude: κ=0.619) while correcting for chance. In terms of classifying HCAT (GP) category, moderate concordance was observed for all models (GPT-3.5 turbo: 64.3%, GPT-4o mini: 69.8%, Claude: 69.2%), with moderate agreement (GPT-3.5 turbo: κ=0.520, GPT-4o mini: κ=0.571, Claude: κ=0.568). Moderate concordance (GPT-3.5 turbo: 60.7%, GPT-4o mini: 66.1%, Claude: 68.1%) and moderate agreement (GPT-3.5 turbo: κ=0.468, GPT-4o mini: κ=0.534, Claude: κ=0.561) were observed for HCAT (GP) stage of care classification. Only GPT-4o mini (74.2%) and Claude 3.5 Sonnet (75.0%) achieved good concordance for classifying patient harm, albeit low agreement (GPT-4o mini: κ=0.162, Claude: κ=0.175). All models performed suboptimally with classifying HCAT (GP) severity, achieving fair accuracy with low agreement.

**Table 3.** Large language model concordance and agreement metrics in complaints classification.

HCAT (GP)^a^ field	GPT-3.5 turbo		GPT-4o mini		Claude 3.5 Sonnet		GPT-3.5 versus GPT-4o	GPT-3.5 versus Claude 3.5	GPT-4o versus Claude 3.5
	Concordance, %	Cohen κ	Concordance, %	Cohen κ	Concordance, %	Cohen κ	*P* value^b^	*P* value^b^	*P* value^b^
Domain	78.4	0.612	79.4	0.623^c^	80.5^c^	0.619	.41	.01^d^	.14
Category	64.3	0.520	69.8^c^	0.571^c^	69.2	0.568	<.001^d^	<.001^d^	.59
Severity	48.8	0.201	53.9^c^	0.226	51.3	0.239^c^	<.001^d^	.03^d^	.02^d^
Stage of care	60.7	0.468	66.1	0.534	68.1^c^	0.561^c^	.38	<.001^d^	.02^d^
Patient harm	57.5	0.114	74.2	0.162	75.0^c^	0.175^c^	<.001^d^	<.001^d^	.25
Average	61.9	—^e^	68.7	—	68.8	—	—	—	—

a HCAT (GP): Healthcare Complaint Analysis Tool (General Practice).

b McNemar test between concordance of AI models.

c These values showcase the highest values within the row.

d Significant *P* values <.05.

e Not applicable.

A comparative analysis was conducted to evaluate the accuracy of HCAT (GP) field classification among the 3 LLMs (showcased in Table 3), aiming to identify statistically significant differences in their concordance with human classification. GPT-4o mini demonstrated superior performance over GPT-3.5 turbo in classifying 3 HCAT (GP) fields (category, severity, and patient harm) with significantly higher concordance rates (*P*<.001) and higher κ values, indicating stronger agreement with human classification. Claude 3.5 Sonnet decisively outperformed GPT-3.5 turbo in the classification of all 5 HCAT (GP) complaint fields, yielding statistically significant higher concordance rates and higher κ values. While comparing between GPT-4o mini and Claude 3.5 Sonnet, we noted that their performance varied across the different classifications (Table 3). Although the 2 LLMs exhibited comparable performance in classifying HCAT (GP) domain, category, and patient harm, GPT-4o mini achieved significantly higher concordance rate (53.9% vs 51.3%, *P*=.02) when classifying severity. This advantage occurred despite having lower κ values, which suggests a nuanced difference in classification agreements. Claude 3.5 Sonnet had significantly higher concordance rate compared to GPT-4o mini in classifying stage of care (68.1% vs 66.1%, *P*=.02) with higher κ values indicating a stronger agreement with human classification.

### Thematic Analysis

Claude’s thematic classification of patient complaints yielded 15 distinct categories (Table 4). The most prevalent concerns were long wait times (n=393, 21.6%), staff attitude and behavior (n=287, 15.8%), and difficulties with appointment booking (n=191, 10.5%), collectively accounting for nearly half of all complaints.

**Table 4.** Thematic classification of patient complaints by Claude 3.5 Sonnet.

	Themes	Values (n=1816), n (%)	
1	Long wait times	393 (21.6)	
2	Staff attitude and behavior	287 (15.8)	
3	Appointment booking difficulties	191 (10.5)	
4	Inefficient processes	159 (8.8)	
5	Technical issues with apps or systems	110 (6.1)	
6	Facility management issues	99 (5.5)	
7	Communication breakdowns	90 (5.0)	
8	Customer service issues	85 (4.7)	
9	Inconsistent medical advice	85 (4.7)	
10	Billing and payment disputes	78 (4.3)	
11	COVID-19–related concerns	72 (4.0)	
12	Accessibility concerns	55 (3.0)	
13	Medication management problems	50 (2.8)	
14	Lack of clear information	41 (2.3)	
15	Privacy and confidentiality breaches	21 (1.2)	

## Discussion

### Principal Findings

Our study analyzed patient complaints using the HCAT (GP) taxonomy, revealing that most complaints (1079/1816, 59.4%) were management related and primarily attributed to institutional processes (994/1816, 45.7%). The 3 LLMs (GPT-3.5 turbo, GPT-4o mini, and Claude 3.5 Sonnet) demonstrated good accuracy in complaints classification, averaging moderate concordance across all HCAT (GP) fields, and high concordance specifically in domain classification. GPT-4o mini and Claude 3.5 Sonnet generally outperformed GPT-3.5 turbo, with significantly higher concordance in several HCAT (GP) fields. Long wait times, staff attitude, and appointment booking difficulties emerged as prevalent complaint themes.

Our study found a moderate complaint rate of 108 per 100,000 attendances per year in 2021, which is comparable to international studies (Saudi Arabia: 780/100,000 in 2019 [23], Ireland: 61/100,000 from 2011‐2016, Netherlands: 64.8/100,000 from 2009‐2019 [24]). The operations department received 56.7% (1029/1816) of the complaints, which aligns with the high proportion of complaints related to institutional processes (830/1816, 45.7%). This suggested that primary care services face challenges in managing administrative tasks, which significantly impact access to care. Analysis using the HCAT (GP) classifications revealed distinct priorities and concerns across different countries. The Netherlands and Ireland reported a high proportion of clinical complaints in their out-of-hours services. In contrast, Saudi Arabia’s complaint profile mirrored ours, with a significant majority related to institutional processes. Comparing our findings to a 1994‐1995 study in Singapore’s primary care sector, which reported a complaints rate of 4 per 100,000 attendances per year [25], we observed that the complaints rate has increased substantially over the years. The increase may be attributed to enhanced accessibility, with two-thirds of complaints submitted online (compared to none in 1994‐1995), and greater patient awareness, with 72% of complaints submitted by patients themselves (up from 46% in 1994‐1995). Globally, the nature of complaints is shifting. In the past, clinical issues dominated [24,26,27], but nowadays, management-related complaints prevail, likely due to improving clinical standards, rising patient expectations [28-30], and evolving health care systems.

The nature of complaints aligned with the harm distribution and LLMs’ thematic classification. Complaints related to long wait times and appointment booking difficulties corresponded to minimal harm, consistent with the HCAT (GP) taxonomy. Prolonged wait times, often exceeding 30 minutes to several hours, are a ubiquitous challenge in primary care practices globally [31,32]. Although typically not resulting in significant harm, these delays can still erode patient satisfaction. In contrast, the single complaint categorized as major harm was attributed to staff attitude and behavior, emphasizing the importance of prompt attention and resolution of patient concerns to prevent escalation [33]. Hot spots consistently emerged during patient encounters, where clinicians often faced time pressures due to brief consultations, mismatched patient expectations, heavy workloads, and cognitive overload, ultimately compromising quality and safety standards [34].

### Accuracy of LLMs in Complaints Classification

The evaluation of the LLMs in classifying patient complaints using the HCAT (GP) framework revealed varied performance across different metrics. We postulated that class imbalance, which was handled differently by each LLM, and differences in the number of variables across fields (domain and severity vs stage of care and patient harm) likely contributed to these discrepancies. Furthermore, inherent differences in LLM architecture, training bias, hyperparameters, and randomness may have influenced results. However, metrics less affected by majority distribution, such as *F*_1_-score, Concordance, Kappa, and McNemar test, provided a better understanding of LLM’s abilities to correctly classify patient complaints. Claude 3.5 Sonnet emerged as the top-performing LLM for complaint classification, achieving the highest *F*_1_-score in most HCAT (GP) fields, highest average highest concordance scores, and statistically significant higher concordance across all fields compared to GPT-3.5 turbo (Tables2  3). In addition, Claude 3.5 Sonnet demonstrated superior performance in 3 out of 5 HCAT (GP) fields, with the highest Kappa statistics in 3 fields. As technology advances and expertise in LLM grows, with newer models being developed and AI assuming a central role in the next frontier of medical research and applications, we can expect future LLMs to achieve even higher accuracy standards. This will reduce the need for extensive model training, thereby decreasing the lag time between development and operational readiness.

Assessing complaints posed significant challenges for both human coders and LLMs. Short complaints were exceptionally difficult to categorize due to their ambiguity and potential overlap with multiple domains. For instance, the complaint “Not reasonable. The time arrangement is not reasonable” (Complaint 21040163) lacked sufficient context to determine its validity. It was unclear whether the issue stemmed from the system’s appointment allocation, prior communication from staff, or the patient’s personal schedule, which would not amount to a complaint in the first place. This ambiguity would have affected the ability and accuracy of LLMs in classifying complaints. Moreover, assessing the severity and level of patient harm in complaints often proved subjective, relying heavily on the words used and the phrasing of verbatim accounts. This made it challenging to accurately determine the category, severity, and true level of harm, as each case was unique. A complaint illustrating this complexity stated, “The attending doctor was impatient and rushed through consultation when I asked about test results” (Complaint 21040245). Human coders and LLMs disagreed on its category classification, with humans categorizing it under “Respect and patient rights,” while LLMs classified it as either “Institutional processes” or “Listening,” subsequently influencing its severity classification. One LLM classified this complaint as causing minimal harm, while another categorized it as no harm, due to the marginal or subtle language used. In reality, complaints often result from a combination of factors, akin to the “Swiss cheese model,” where systemic issues, such as inadequate consultation time, contribute to downstream unprofessional behavior [35] or potentially compromising patient safety. While all classifications had some merit, achieving 100% accuracy may be unrealistic. Further training, dataset refinement, and allowing multiple classification selections may enhance LLM accuracy, but context-specific fine-tuning would still be necessary. Usage of newer and different LLMs such as DeepSeek and QWEN can also be considered to enhance accuracy in complaints classification.

While the majority of patient complaints fell within the 3000 character limit set for our LLMs, we had to truncate a small percentage (2.6%) to ensure compatibility with historical LLM token length limitations (GPT-3.5’s 8000 input token limit). While current technology allowed for longer input lengths, we maintained this truncation approach to ensure the system’s robustness in handling arbitrarily long complaints in real-world applications. Future studies could explore strategies for handling longer complaints without truncation.

Our approach to assigning ground truth labels based on the most viable classification for each complaint may have implications for complaints with multiple issues. While this approach aligns with our current process for handling and labeling patient complaints using the HCAT (GP) classification, which focuses on identifying the root cause, it may not fully capture the complexity and multitude of factors contributing to the complaint. Notably, our preliminary results showed that allowing the LLM to output multiple possible labels increased accuracy but compromised our ability to identify the primary issue. This trade-off highlights a potential limitation of our approach and suggests that future studies could explore the benefits of multilabel classification, which may provide a more nuanced understanding of complaints with multiple issues.

### Strengths and Limitations

Our study has several notable strengths. We conducted a comprehensive analysis of complaints from an entire public primary health care cluster in Singapore, comprising 7 clinics, multiple departments, and stakeholders. We used the well-established HCAT (GP) taxonomy, facilitating comparisons with international studies. Furthermore, we were one of the first few who studied the application of LLM in classifying patient complaints using the HCAT (GP) framework and used a robust evaluation metric framework to assess LLM performance. However, we acknowledge potential biases in human coding, which we mitigated through consensus discussions. Nevertheless, individual coders’ experiences and perspectives on the health care system may have influenced complaint classification.

Our study has several limitations. First, our figures on the number of complaints per visit may be subjected to overestimation, as we only accounted for physician attendances, excluding nonmedical staff interactions. The retrospective study design, using 2021 data, may also limit generalizability, as many complaints were related to COVID-19 or postpandemic issues. This may not accurately reflect current complaints, highlighting the need for ongoing data collection to ensure the robustness of the LLMs studied. In addition, we used pretrained LLMs without fine-tuning, which may limit their performance. Patient phrasing and biases may also influence complaint classification. Limited sample sizes in certain categories, such as major harm, may restrict the robustness of statistical analysis, but given the zero-shot classification approach, this limitation may not directly impact the reliability of the LLMs’ classification performance. Furthermore, our study relied on predefined categories and allowed only one variable per field, which may oversimplify the complex, multifactorial nature of patient complaints. The undisclosed training data, varying model sizes, architectural differences, and distinct zero-shot learning capabilities among LLMs likely introduced inherent biases and limitations that affected their baseline accuracy in classification tasks, which were beyond our control to detect or adjust for in this study. Language differences, including “Singapore English,” may also affect LLM classification. Moreover, our study only included formal complaints [40], and future studies may benefit from incorporating social media reviews. Future studies could also compare LLM classification to traditional statistical machine learning models and BERT-based models, which are valuable comparators given their established performance benchmarks, interpretability, and widespread adoption in NLP tasks.

### Conclusions

In conclusion, our study provides valuable insights into patient complaints in primary care settings, highlighting the prevalence of management-related issues and the potential of LLMs in complaint classification. GPT-4o and Claude 3.5 have demonstrated promising results in complaints classification in accordance with the HCAT (GP) taxonomy, but further fine-tuning and model training are required to improve accuracy. Claude 3.5 Sonnet showed particularly strong performance. LLMs can practically be applied in health care organizations to support service quality staff in prioritizing complaints, escalating high-risk ones to senior staff, and distributing minor complaints to individual specialties for handling. The findings have implications for health care quality improvement and patient safety, and future studies could involve enhancing LLM performance and integrating LLM into complaint analysis to facilitate proactive identification of systemic issues.

## Supplementary material

10.2196/74231 Multimedia Appendix 1 Jupyter Notebook code file.

## Abbreviations

AI: artificial intelligence
GP: General Practice
HCAT: Healthcare Complaint Analysis Tool
JSON: JavaScript Object Notation
LLM: large language model
NLP: natural language processing
